# Developing more inclusive approaches to animal research and patient involvement

**DOI:** 10.1007/s11019-025-10288-1

**Published:** 2025-08-13

**Authors:** David Mawufemor Azilagbetor, Gail Davies, Lester Darryl Geneviève, David Martin Shaw, Bernice Simone Elger

**Affiliations:** 1 Institute for Biomedical Ethics, University of Basel, Basel, Switzerland; 2 Division of Clinical Psychology and Psychotherapy, Faculty of Psychology, University of Basel, Basel, Switzerland; 3 Geography Department, University of Exeter, Exeter, UK; 4 Faculté de Médecine, Université Laval, Québec, Canada; 5 Care and Public Health Research Institute, Universiteit Maastricht, Maastricht, The Netherlands; 6 Center for Legal Medicine, Unit for Health Law and Humanitarian Medicine, University of Geneva, Geneva, Switzerland

**Keywords:** Animal experimentation, Democratic approach, Patient involvement, Research ethics, Research policy

## Abstract

Doing scientific research with animals is a subject of intense societal debate, often involving polarized and public discussions with stakeholders and groups interested in animal research. Patients, given their medical conditions, have a high stake in biomedical research, including research involving animals. However, their perspectives are rarely heard in policy-related discussions on animal experiments. This essay discusses the positions and stakes of groups involved in public discourse and policy-relevant engagements. It further explores the legitimate interest of patients and the need for an all-inclusive approach to animal research policy. This subject is complex and democratic societies must address societal issues with an all-inclusive approach to reach policy decisions reflecting the interests of all stakeholders. The positions of groups—pro-animal research stakeholders and anti-animal-research advocates—with vested interests involved in animal research discourse considerably shape research policies. Animal research policies arguably affect patients. Through democratic ideals, inclusive approaches that are suitable for resolving science-driven societal issues, and initiatives currently guiding animal research policies, patients need to actively be involved in public discourses and policy-relevant decision-making processes in deciding the place of animal research in biomedical advancement as a society.

## Introduction

Doing scientific research with animals is a subject that has seen intense debates over centuries (Franco 2013). There have been significant changes in its landscape over the years but it still remains controversial and a relevant subject of ethical and societal discussions. Public debate on animal research influencing policies and regulations is generally led by various voices, depending on their specific objectives and goals. These include voices communicating about animal research through public engagements and openness and transparency activities (Speaking of Research 2012, 2021; McLeod and Hobson-West 2016), voices of advocates of alternatives to animal experimentation, as well as voices of advocates of animal rights and protection opposing animal research (Pugh 2016; National Anti-Vivisection Society 2017; American Anti-ViviSect. Society 2024; Animal Aid 2024; Humane Society International 2024). But these are not the only groups with interest in animal research. As a societal matter, the perspectives of all stakeholders and interest groups are needed for consensus-based decision- and policy-making. By using “consensus” in this essay, we refer to the involvement of all stakeholders in finding the most agreeable and workable policies in specific research contexts that can guide future scientific research. A major section of society whose perspectives to consider in these discussions is that of patients—people affected by health conditions. Owing to the current rationale for animal research–beneficial outcomes—patients can be cited as the beneficiaries of animal research. In this regard, their perspective in the animal research political terrain is important to consider in current and future policies and legislation. Considering the perspectives of patients is needed in addition to all other involved stakeholder groups to find societally acceptable resolution to the place of animals in biomedical research.

Scholarship in patient involvement and engagement in medical and health research has been gaining ground over the past years (Gibson et al. 2012; Mathie et al. 2014; Ocloo and Matthews 2016; Liabo et al. 2020; Turner et al. 2020), leading to scholarly work on patient involvement and engagement in research matters that are further upstream from the clinical studies commonly understood to concern patients directly (including animal research which mainly falls under preclinical research). Major scenarios in which patients or their representatives encounter animal research include their review of research projects (some of which involve animal studies) in funding schemes and visiting animal research facilities where animal experiments are conducted (Cowan 2018; Gorman and Davies 2019, 2020). Meanwhile, a further step is necessary to place patient encounters with animal research at the societal and political level. The stakes for patients in the public debate are high, given their immediate interest in the outcomes of research conducted with animals, but the lines between patients and the public are blurred and the terminology is often contested. As a result, more has to be done to consider carefully the patient involvement in relation to other voices leading discussions in this area. Animal research brings forth societal concerns, and society as a whole needs to collectively determine how animals are used in research. Political decisions that ensue from public discourses that do not include patient interest groups will not be representative of society (Feeney 1987) or the interests of publics as current or future patients. In this essay, we discuss societal positions on animal research and interest groups leading policy-relevant debates, followed by the elucidation of patients’ common interest in biomedical research including research involving animals and why their voice matters. Subsequently, drawing generally on the Swiss context, we construct the rationale for legitimate and meaningful patient involvement in policy-relevant animal research discussions and decision-making. We then discuss how patient involvement in animal research can be approached in practice whilst addressing the challenges such involvement faces.

It is necessary to highlight that the use of the term “patient” is contested, especially when it comes to public involvement and participation in healthcare matters. Some argue that “patient” denotes a suffering individual who longs for relief from the services of healthcare professionals in an unequal relationship (Neuberger and Tallis 1999). Here, the word denotes the passivity of the patient who is supposed to take an active part and responsibility in their health care. It is also argued that other healthy individuals seeking health services do not fit in this notion of the patient since they only encounter health professionals to discuss choices and for counselling (Phelps et al. 2008). Alternative terms like "user", "client", or "consumer" are therefore proposed (Neuberger and Tallis 1999; Hutchison 2006). Others however argue that the term "patient" still describes the modern definition of a health service user who is an active participant in their healthcare (Neuberger and Tallis 1999), and there is no satisfactory alternative term (Hem 2013). The view of patients themselves on this contestation is divided and what exactly they want to be called is inconclusive (Nair 1998; Deber et al. 2005; Costa et al. 2019). The terms “consumer” may be used for people taking up involvement roles in research endeavors (Arain et al. 2015), but the term “patient” also figures in this context’s literature (Harrington et al. 2020). We will use the universal term “patient” in this essay as its use is deemed appropriate since there is no agreement and conclusion about the exact appropriate or patients’ preferred term (Costa et al. 2019).

## Animal research, varying positions and stakes

Animal research as a subject of societal concern is grounded in varying positons, opinions and interests. As a human society, there is arguably some sort of a general societal agreement to pursue biomedical science and research to advance knowledge for biomedical progress. But, when it comes to the use of animals for this purpose, considerable disagreement emerges. Generally, scientists use animals in research to study and understand disease mechanisms and find cures for health conditions. But they are often questioned regarding the legitimacy of using animals for such purposes (Porter 1992). Arguments for and against researching with animals constitute two major positions that are often in direct opposition, with each side providing arguments to support their respective positions (Bateson 2005).

Agreement and disagreement about animal research are embedded in ethical perspectives and philosophies. One reason that animal research is controversial is because of the existence of different ethical standpoints regarding animals, and their use in research. Different principles can be evoked to justify both sides of the debate—the rightness and wrongness of animal research (Bateson 2005). Utilitarian arguments, for instance, form a major group of arguments used to justify the use of animals in research. The utilitarian principle deems an action ethically justifiable if it results in the greater good for the greater number involved—generally, society at large (Mill 1871; Bentham 1890; Niemi 2019). Through this view, which is widely applied in animal research through a ‘harm–benefit analysis’ concept (Kalman et al. 2010; Griffin et al. 2014; Brønstad et al. 2016), it is possible to justify animal research by arguing that the beneficial outcomes of the experiments for society at large justify the use of the animals, demonstrating that the suffering of animals can only be acceptable insofar as it results in societal benefits (Griffin et al. 2014). Nevertheless, it can be observed that this argument depends largely on the status given to animals to permit using them as means to obtain human ends. This observation brings us to one of the major opposing arguments against animal research—the moral status of animals. Moral status of animals is a complex philosophical subject, which is still debated without concrete conclusion or consensus (DeGrazia 2002; Gruen and Monsó 2024). Some argue for intrinsic moral status of animals that makes them deserving of rights, life and better moral treatment by humans (DeGrazia 1991a, b; Franco 2013). This underpins the animals’ rights positions held by many that questions the ethical permissibility of animal research.

Furthermore, for the past several decades, animal research debates have shifted from their basis on animal ethics towards the scientific utility of animal model research (Akhtar 2015; Balls 2021) creating some division in the research community. Whilst many scientists still recognize its relevance and indispensability for scientific and medical progress, another section of scientists argues against animal research. A significant argument in the scientific community, for instance, is one related to the lack of reproducibility of animal research results (MacLeod and Mohan 2019; Wilson et al. 2023). Other limitations have to do with the lack of translatability of animal research into clinical human health benefits (Pound et al. 2004; Bracken 2009; Ioannidis 2012). Some interpret this as a result of poor reporting, poor experimental designs and statistical analyses, and over interpretation of results among other problems (Collins and Tabak 2014; Begley and Ioannidis 2015; Green 2015; MacLeod and Mohan 2019; Wilson et al. 2023). Others deem this concern the result of the failure and ineffectiveness of animal experiments in contributing to human medical progress (Pound et al. 2004; Akhtar 2015). The divided positions in the scientific community render the subject even more complex as it can be observed that groups against animal research, whose opposition to animal research is mainly grounded in animal ethics, also use some of these arguments to support their position that animal research is unethical as it is needless, scientifically questionable, and does not contribute to human health benefits.

The extreme ends of the spectrum of animal research debates are comprised of the voices of its supporters and its opponents. In this regard, supporters of animal research may have some vested interests (Balls 2021). Michael Balls, a biologist who has worked extensively on animal research policy and advocates for a shift towards alternative methods, fears that defending animal research in support of vested interests is one reason why recognition of the concerns of animal research and the search for new approaches in biomedical research are limited (Balls 2021). These vested interests can question the arguments made regarding the utility of animal research. In describing the defense of animal research, Balls noted that supporters of animal research have "turned more and more to the use of counter-propaganda, rather than providing sound scientific evidence" in the face of science-based criticisms against animal research (Balls 2021). The thought of researchers having vested interests when it comes to public discourse about animal research is also heard anecdotally among voices opposed to animal research. Meanwhile, at the other extreme end of the spectrum, we have the opponents of animal research. The opposition group is quite diverse, but it is mainly dominated by animal rights and welfare protection advocates and organizations, whose objective is said to be about raising awareness and challenging conventional human-animal relations (Munro 2005). Even though individual members may hold diverse viewpoints and have diverse objectives regarding laboratory animal research (Plous 1991), a major concern is based on the harm imposed on animals, and the main goal is to liberate animals and stop animal research (National Animal Interest Alliance 1993; Holder 2014; Animal Charity Evaluators 2024). There are discussions about the need for a turn in the approach of the animal rights movement toward the use of deliberative democratic structures to attain their objectives (Garner 2017, 2018, 2019; Parry 2017; Spang 2022). Their interest in animals used in research would therefore mean more involvement in policy-making processes regarding animal research.

In the midst of all these concerns about the voices involved in animal research policy debates, these discussions and debates are significantly influenced by interests. As a subject that attracts different views and reactions from different spheres of society, observing the expression of interests in public discussions is to be expected. Nevertheless, the main issue here is that, as much as it can be observed that interests fuel the discussion, the voices of patients are more rarely involved in the expression of interests. Patients are the people affected by the health conditions studied by the means of animal experiments. Therefore, they have a legitimate interest, but their perspectives are not heard in these discussion at all, or in the very least, not as much as the other two groups of interests to result in policies that take into account the interests of all stakeholders. When patient interest are presented, they are also often represented by others, forming part of propaganda either promoting or contesting animal research (Davies et al. 2024a). Why should they not be involved in public discourses and policy-relevant discussions and processes with the rest of the stakeholders to inform animal research policy?

## Societal-inclusive approach to animal research

Currently, the general approach to animal research policy puts emphasis on replacing animals in experiments as much as possible, and endeavoring to develop and expand alternative methodologies to animal models to completely replace animals in science as soon as possible. In this case, the status of scientific evidence and progress (in, for example, the performance and validation of alternative methods) will be instrumental in guiding policy development in this area. Nevertheless, a democratic approach has been driving animal research policies, especially in some democratic contexts. In democratic practices, decision-making power is vested in the people. In the context of animal research, the general public has been shaping animal research through the power vested in them.

Involving the public directly, some systems take a democratic approach to make a societal decision on the current and future use of animals in research. In 2022, the Swiss public, through a popular initiative, went to the polls for the fourth time to reach a societal decision on the continuity or phase-out of all animal experiments (von Roten and Bauer 2022; The Federal Council—Swiss Government 2022). Furthermore, in the European Union, citizens’ initiative dubbed “*Save Cruelty Free Cosmetics—Commit to a Europe Without Animal Testing*”, after garnering more than one million signatories, was submitted to the European Commission in 2023, asking for a significant decision on some uses of animals in safety assessment, to a large extent, asking the union to consider modernizing science by committing “to a legislative proposal plotting a roadmap to phase-out all animal testing in the EU before the end of the current legislative term” (European Citizens’ Initiative 2023). Additionally, regulatory evaluation and authorization of animal experiments in many jurisdictions around the world use democratic approaches, involving the inclusion of different views and perspectives of society. With this approach as the basis, oversight bodies are constituted in such a way that members with different opinions foster a dialogue in which they initially hold conflicting views and interests but eventually have to work together to jointly define what is and is not acceptable in terms of animal experimentation on behalf of society. For example, in the Canton of Fribourg in Switzerland, the oversight body includes, representatives from organizations whose statutory aim is the protection of animals, scientists from institutions carrying out animal research, a veterinarian, and a member who is trained in law or ethics (Règlement sur la protection des animaux—Etat de Fribourg—Recueil de la législation (RSF 725.^11^) 2024). The accountability of these bodies and the transparency of their decision-making is also largely explored from the lenses of the public and society as a whole (Azilagbetor et al. 2024).

Even though societal concerns still remain, animal experimentation is currently regarded as permissible, mainly due to a general societal acceptance, as observed from public opinion polls and referenda in the Swiss context, (von Roten and Bauer 2022; The Federal Council—Swiss Government 2022). This general societal agreement has not only permitted animal research; it has also shaped animal research, collectively determining which animal experiments are ethical and acceptable and which ones are not. Research into societal stances uncovers issues of which animal species should be used in research, which are not, and for what particular purposes research should be done on animals. A general debate on the acceptability of research with great apes (Aguilera et al. 2021) led to the restriction of the use of chimpanzees in biomedical research in the USA (Chris Adams 2013; Reardon 2015), the EU (European Commission 2010), and other countries (Project R&R (Release Chimps) 2023). This is believed to be a reflection of public support for a ban (Thew et al. 2012). This decision led to the reduction of funding for chimpanzee research with public funds. The NIH, for example, withdrew support for chimpanzee research in the USA (Collins 2015). In addition to the sorts of animal species to be involved in research, societal agreements also determine animal research objectives where the use of animals for certain benefits has been deemed unacceptable. For instance, animal research for non-medical and cosmetic purposes is generally deemed illegitimate, leading to its ban in several countries around the world (Sreedhar et al. 2020).

## Putting patients in an all-inclusive societal approach

To adequately address all concerns regarding animal research, more agreeable policies need to be developed, and this requires the inclusion of all views and perspectives, in addition to the positions of every stakeholder of society in the process. Developing legitimate animal research policies in this regard necessitates including patients in the process because they are the stakeholder group that expects the most immediate benefits from animal experiments.

Some authors have also discussed taking further democratic societal approaches in addressing the dilemma of animal research (Garner 2016; Khoo 2018). Khoo (2018), for instance, proposes a democratic approach that involves public input regarding specific disease areas that should be considered in ethical animal experiments. A democratic approach would involve the engagement of all public stakeholder groups who have an interest in animal research. Looking further, specifically, this suggestion about considering disease areas concerns mainly patients as they are the group affected by health conditions. This again highlights the important place patients need to hold in animal research policy development through democratic and societal consensus-based resolutions. In consequence, any discussion about animal research with stakeholders should go hand-in-hand with the consideration of the involvement of people affected by health conditions.

Engaging societal stakeholders can also help resolve a major stalemate which concerns the need for animal research in biomedical progress. It can be observed that evidence used in debates about the usefulness or ‘uselessness’ of animal research is contested. Researchers have long argued over the evidence (Olson et al. 2000; Lindl et al. 2001; Bakhle 2004; Blakemore and Peatfield 2004; Pound et al. 2004; Knight 2008; Clark 2015; Monticello et al. 2017; Bailey and Balls 2019), making the facts about the actual role of animal research uncertain. Moving further from here, political pressure continues to mount on policy-makers to respond to public concerns regarding animal research. In some instances, milestones are envisioned and deadlines are demanded to speed up the transitioning of animal experimentation into the ideal status envisioned by some interest groups—complete replacement within specific time frames (European Citizens’ Initiative 2023). These demands and expectations do not only call for a rapid response, but also point out the urgency of the matter and the need for a societal resolution. These peculiarities—societal (i.e., values being in conflict), political (i.e., urgent decisions being demanded) and scientific (i.e., facts being in discord)—in the landscape of animal research are comparable to those in complex science-policy-related issues posited by Funtowicz and Ravetz (2003) who stated that “in the sorts of issue-driven science relating to the protection of health and the environment, typically facts are uncertain, values in dispute, stakes high, and decisions urgent”, in their theory of post-normal science (PNS). In situations with this sort of attributes, PNS proposes a different approach to decision-making and problem-solving which relies on community and stakeholder engagements, rather than traditional reliance on expert knowledge.

As public concerns are characterized by values and emotions, which tend to populate political resolutions to animal research policy and regulation, an approach based on PNS, which involves the engagement of all stakeholders involved, will be helpful in solving the issue. When PNS is applied to resolving complex issues like this, it is believed to provide a consistent framework that makes way for expanded participation in decision-making. Instead of traditional expert engagement, PNS solves complex societal problems of science and philosophy by bringing facts and values, where its “principle of the plurality of legitimate perspectives on any problem leads to a focus on dialogue, and on mutual respect and learning, wherever possible" (Funtowicz and Ravetz 2003). In effect, as the PNS framework appeals to a wider public engagement in resolving societal issues, its emphasis on the need to consider the plurality of legitimate perspectives, dialogue, and mutual respect also appeals to a greater involvement of patients as concerned members of society in resolving the societal concerns with animal research. In this regard, the need for more patient involvement and engagement is again highlighted as an imperative in an all-inclusive approach to legitimate societal agreement about the place of animals in biomedical research now and in the future.

## A case for patients’ interests

The general demonstration of the benefits of animal research suggests that current and future persons who benefit from medical treatments or other forms of health services are the principal beneficiaries (Committee on the Use of Laboratory Animals in Biomedical and Behavioral Research 1988). The possibility of relieving the pain and suffering of patients has been cited as a solid ethical justification for animal experiments, even in the ones in which harm is caused to animals (DeGrazia 1996; Ideland 2009; Wayne and Glass 2010). And when they discuss in favor or against the usefulness of animal research, the major voices involved in animal research discussions generally place patients at the center of their cases in reference to the aims of biomedical research (Hewitt 1990; Rowan 1997; Greek and Greek 2010; Jucker 2010; Green 2015).

Furthermore, the medical interests of patients demonstrated by numerous health benefits (Committee on the Use of Laboratory Animals in Biomedical and Behavioral Research 1988; Domínguez-Oliva et al. 2023) and other societal benefits of animal research e.g. knowledge gain (Tannenbaum 2019) are one of the main criteria that justify animal research. Through the concept of harm-benefit analysis—weighing animal interests against human and environmental interests—animal research is only justified when applicable benefits result from it (Brønstad et al. 2016), and patients tend to be the most frequently cited direct benefactors, even around pre-clinical research.

Just like all other voices in animal research discussions, patients also have a stake in animal research; "patients have a particular interest in research about their specific illness.” (Kalman et al. 2010). This is particularly noted in the need to involve patients in regulatory evaluation and authorization of animal research protocols where it is considered that just “like animal protection organisations, they represent a part of society with a particular interest in animal research where their voice can be helpful in evaluating experimental protocols” (Kalman et al. 2010). The general interest of patients is grounded in the fundamental reason for animal research—the need to advance treatments of their health conditions. This interest can arguably be considered the supreme interest of all other interests presented above, but for the sake of the need for all groups to reach a mutual ground, it should be agreed that this interest should be placed side-by-side with the interests of the other stakeholders involved in public and policy discussions.

It should be noted that highlighting the interest of patients is grounded in the overall rationale for biomedical research should not be interpreted that this interest is a de facto justification for animal research. It may be mentioned, especially by those who are of the opinion that animal research does not contribute to medical benefits, that it is exactly for the benefit of patients that animal research must not be pursued. This is because, if the goal of biomedical research is to advance medical treatments and animal research does not advance this goal, but rather produces research results that lead to opposite outcomes of this goal (Akhtar 2015; Green 2015; Archibald 2018a; Balls 2021) (and probably leads research efforts away from reaching the main goal), then it should not be practiced; alternatives to animal research which will offer much benefits should be preferred (Akhtar 2015). However, the goal of biomedical research (whether it involves animals or not) remains the same and so is patients’ interest—to obtain health benefits through biomedical research. Research on patient and public involvement and engagement (PPIE) carried out by Davies et al (2024b; 2024c; 2025) in the UK demonstrates some researchers have found ways to incorporate patient experience and expertise in making decisions about translational research using animals, such as around research focus or animal model choices. This, as a result, further highlights why patients need to have a say in finding societal consensus regarding the methods of biomedical research, with or without animals, to advance health benefits.

Additionally, this conversation can be taken to the level of basic research which also sees significant animal research as compared to translational research. It could be argued that basic research only generates knowledge and might not be relevant for patients to warrant their involvement. Indeed, knowledge gained from basic biomedical research using animals would have to go through various translational stages before reaching the clinical application stage to benefit patients. However, first and foremost, it is from basic research that clinical research and health benefits emerge. So there may be no benefit for patients in clinical needs that still need to be met from animal-based basic research. Further, with the advancement of medicine, healthcare is becoming more and more personalized and patient-centered, and benchside biomedical research is becoming directly applicable to its end-users at the bedside. Patients are now getting closer and closer to preclinical research, and research involving animals could be directly applicable to the treatment of groups of patients waiting purposefully for such specific therapies. An example is the development of targeted cancer therapies for patients by the use of Patient-derived Xenograft (PDX) models, where cancer tissues from patients are implanted into animal models in preclinical studies (Abdolahi et al. 2022; Liu et al. 2023), advancing personalised and patient-specific animal research. Patients are also being involved in targeted research for specific disease areas which involves animal (Cowan 2018). As a result, the view that animal research is not patient-facing might be totally untrue. This example portrays how patients benefit directly from studies conducted in animals, and how patients are now closer to preclinical research more than ever before, demonstrating the magnitude of their interest in animal research and the need for their involvement in political discourse.

## Limited involvement of patients

There exists little to no involvement of patients in policy-relevant discourses. The fundamental barrier to their active involvement could be that patients are not adequately engaged and invited to the decision-making table, as noted by Denis Feeney (Feeney 1987). (It should be noted that this account is on someone with a patient perspective but who is also expressing the same in a particular political and healthcare setting, the USA.)

When the 1980s saw a turning point for the subject of animal research leading to the development of significant national and international regulatory policies (Council for International Organizations of Medical Sciences (CIOMS) 1985; Interagency Research Animal Committee 1985; US Policies and Laws 1985; Council of Europe 1986; European Economic Community 1986; Holden 1986; Legislation UK 1986) that aimed to address such concerns, Feeney, a paraplegic patient, thought that the ensuing policies taken around that time had little consideration for the interests of patients. Feeney mentioned that “in the debate over the use of animals in research, we in the fraternity of the infirm have been almost completely ignored”, while highlighting the potential consequences that such a neglect could have on patients hoping for medical advancements to address their health conditions (Feeney 1987). Being part of an interest group with a major stake in animal research, Feeney presented a few ensuing consequences of them not being represented on national inter-interest group committees where deliberations took place to inform significant animal use policies: the training of “monkeys to assist quadriplegics (similar to the way in which seeing eye dogs assist the blind)” was condemned, and the National Institutes of Health (NIH), without prior consultation with patients suffering from brain injuries and their families, withdrew all federal funding for an important head injury research despite the fact that the Head Injury Foundation of America actually wanted the work to continue (Feeney 1987). Indeed, the lack of the engagement of patients in decision processes results in their exclusion from discussions that lead to significant political and regulatory decisions.

Further, another reason why patients may not actively be engaged could be that they may be torn by the potential conflict between their moral stances on treating animals ethically and their desire for the treatment of their diseases. Patients who are conflicted between the welfare of lab animals and the alleviation of their suffering may stay away from animal research discussions altogether. These patients would belong to the group of people who remain ambivalent in public animal research discussions, as found by Lund and colleagues (Lund et al. 2012, 2014).

Further still, the sensitivity of the animal research subject could be another factor. Due to the polarity of debates, it may possibly be daunting for patients to be involved in the discussions. Moreover, current engagements with animal research being "dominated by the highly emotive campaign materials used in animal rights activism," can be a barrier to the engagement of everyone —patients among other stakeholders —in animal research matters. Such campaigns may occur in a highly charged sensory environment for people already struggling with health to engage (Davies et al. 2020). Additionally, patients may feel guilty for being part of the reason why research that a part of society dislikes has to continue, and they may avoid discussions where views are highly polarized.

It is also possible that patients are not engaged because of limited technical expertise and medical knowledge which may prevent them from meaningfully engaging in discussions around preclinical animal research. Let us take cancer medicine for instance, where patients with limited medical knowledge may not be able to meaningfully discuss the role of preclinical trials for a potential cancer therapy. Limited expertise may also hinder their involvement in downstream animal use at the various points in medical advancement and health care. While these limitations and challenges remain, patient involvement in animal research discourse remains important in developing its policies, and these challenges need to be circumvented to foster the engagement of patients.

## What does patient involvement add to the all-inclusive approach?

A crucial point to highlight is that attempts to involve patients in animal research discussions should not be regarded as a path that will feed in emotions to either further general societal acceptability of animal research or overlook animal welfare issues related to animal experimentation. Rather, the rationale is to ensure that discussions about biomedical research with animals involve all societal interest groups and that policy decisions that are made are more representative of society.

There is no doubt that not all patients would support animal research. In the past some patient organizations were formed to counter the advocacy of pro-animal research patient groups which used patients stories to advocate for animal research (Davies et al. 2024a). It is also possible that patients with opposing perspectives on animal research firmly hold animal protection views and disapprove of animal research rather than inclining towards their tendency to advance their personal health interests by supporting animal research. Nonetheless, as stated earlier, the rationale for patient involvement in policy discussions should underpin and welcome the expression of mixed perspectives of patients. Whether they have different views on animal research or not, their common interest would be to support endeavors that find solutions to health conditions, and this supports the need to involve them in order to consider their variable perspectives on animal research. Those who do not support animal research may hold such views around what they think should be done to meet their needs, for example by advocating for the use of alternative methods. Also, since the discussion is about how animal research needs to be approached considering diverse societal views, the varying positions of patients should not be considered an issue. If genuine engagement of patients’ views is the goal, their differing positions need to be managed and incorporated into the process. Moreover, even though not all patient groups would support animal research, their various positions are all needed in this societal approach. Their different positions will open ways of finding mutually accepted means of reaching the same goal—finding treatments for health conditions.

There might be an entrenched tendency to think that the interests of patients (considered in this case as people for whom other voices can speak) are expressed by expert interest groups like scientists or clinicians, who communicate the importance of animal experimentation in policy-related discussions. We highlighted above that the involvement of scientists in animal research discourses could be marred by their vested interests and subsequent defense of animal research in some cases. But let us also imagine that it is true that the engagement of animal researchers in policy discourses, to some extent, is intended to advance patients’ interests. Nevertheless, these voices speaking on behalf of patients may be limited. Involving non-expert groups in the framework of PPIE is important and is currently highlighted as such. Patients and members of the public are said to offer distinctive perspectives on policy-related issues based on their own practical experiences, which expert knowledge may not provide (Aiyegbusi et al. 2023). Non-experts are therefore urged to be engaged in regulatory science and policy-related processes that directly or indirectly affect them (Aiyegbusi et al. 2023). Patients are unique in research matters, carrying their own opinions which they can contribute to discussions. According to this view, involving patients can help refine and clarify the human benefits of animal research. Concerns are raised about the expected benefits of scientific research, especially in the context of biomedical research where end users are far from the laboratory and it is feared that research does not frequently reach the clinic (Khoury et al. 2007; Collins 2011). With patient involvement, we can reconsider current approaches to doing medical research by hearing what is important to patients in the context of animal research and translational research. For instance, considering their experiences with their health conditions as well as with medical research in general, patients may provide insight into translational medicine where preference for a human-based approach to biomedical research (Archibald et al. 2018b) instead of animal model-based research may be explored. Patients live with health conditions and their experiences can inform the choices of research approaches that can best address their conditions. Questions regarding efficacy, fitness for purpose, urgency of treatment, and cost-effectiveness may also be explored in context by listening to patients.

Furthermore, patients could be categorized in the same group with members of the public—a non-expert group—in research matters, as in the case of the PPIE framework, for example. This could result in another tendency to consider patients as members of the general public or lay members, some of whom may already be actively involved and leading animal research discussions. Yet, in the medical sciences, patients, for many reasons, including their lived experiences with diseases, are distinct from healthy members of the public. Just as members of the lay public (here, “members of the lay public” comprises patients and the general public) bring contributions that expert knowledge may not provide in policy-related discussions, patients in *stricto *sensu can also bring on their unique experiences and contributions to policy-related engagements that may not be acquired from members of the general public (Shaw and Elger 2014). In essence, the concept of ‘a patient’ should not be generalized to a healthy member of the general public in this context. Meanwhile, given that anyone could become a patient at any time, it would be expected that all voices (i.e., healthy people) involved in animal research discussions would consider including interests of their future ‘*patienthood’* in their discussions. We cannot be sure whether these voices leading the discussions actually consider the days of their *'patienthood'* in their debates or not. But what can be pointed out is that ‘real’ patients are represented by various organisations to advance their interests and advocate for their special needs, which includes conducting research into their conditions. The only way to be certain about the full expression of patients’ voices is to include ‘real’ patients (i.e., people currently affected by health conditions) in discussions. Therefore, since ‘*patienthood’* and an interest in biomedical research borne out of a patient’s experience with a health condition cannot not be generalized to the other voices leading animal research discussions, the representation of the unique voice of patients is needed.

## Opportunities for involvement of patients in animal research policy efforts

### Patients in ethical oversight of research

Efforts to involve patients in ethical discussions of research endeavors in order to represent their own interests have been gaining ground. There have been calls for many years for patients to be involved in human research ethics committees (RECs) to have their say in study reviews (Shaw and Elger 2014; Rakic et al. 2017). Animal research is also fundamentally a subject that falls under research ethics, and as patients have interest in it, they should also be involved in ethical discussion here. Involving patients in ethical oversight of research opens opportunity in the animal research landscape as well, and patient involvement in animal research ethics committees should be considered. This aligns with Kalman and colleagues’ (2010) point about patients’ involvement in animal research ethics committees: “they are able to express opinions about aspects of therapy of their disease".

However, it is important to recognize that people bring more than their lived experience of health conditions when evaluating animal research. Gorman and Davies conducted interviews with people reviewing biomedical research proposals using animals for medical research charities. They found patients were interested in research on specific diseases but also on other aspects of animal research such as harm and societal concern. People affected by health conditions wanted to know more about the harm aspects of experimental procedures, the application of the 3Rs, and ask questions about researchers’ prior consideration of alternative methods before proposing the use of animals to answer their research questions (Gorman and Davies 2020).

Patient representatives are already carrying out an informal ham-benefit research, in seeing “themselves as connecting societal concern for animal welfare with their personal interest in realising benefits from research” (Gorman and Davies 2020) when reviewing animal research projects. There are things to learn from these specific and case-by-case evaluations of animal research projects where patients are already making meaningful contributions to ethical evaluation and authorization of animal research projects. With patients completing the interest groups involved in oversight bodies, collective decisions made by these bodies on each animal research project can be considered more inclusive of all interest groups and representative of society.

### Opportunities in openness and transparency initiatives

Public communication about animal research led by major stakeholders has been improving over the years. Researchers have come to understand the need to be open about the use of animals in research and are encouraged to engage in public and political communications about animal research (Clark et al. 2019). Openness and transparency have become a recognized approach to open dialogue and societal engagement on the subject of animal research. In the UK, the *Concordat on Openness in Animal Research* (Understanding Animal Research 2014) initiative enjoins research institutions to be open and discuss the use of animals in their research programs. In Switzerland, the *Swiss Transparency Agreement on Animal Research (STAAR)* was initiated in 2022 to bring together public and private institutions that conduct, participate in, or provide funding for animal research, as well as establishments that breed and supply animals (Swiss Universities 2024). One of the main commitments of the signatories of this agreement is to enhance their communication with the public and the media about their involvement with animal research. The agreement's standards are elaborated each year. One of the 2024 objectives is dedicated to public engagement approaches, and signatories are committed to organising public events, including debates and open days where the public can learn more about their animal use and interact with them (Swiss Universities 2024).

Animal research may not be patient-facing, but they can take advantage of these initiatives and be actively involved in the discussion. These transparency initiatives can involve societal groups and stakeholders interested in the subject of animal research and offer an opportunity to have rich discussions involving diverse opinions. Patients can also be involved to have significant discussions among other stakeholders about the role of animals in medical research and current realities discussed by both researchers and other voices in society.

### Involving patients in national policy- and decision-making processes

As conveyed by Feeney (Feeney 1987), political decisions on animal research that ensue from public discourse that excludes patient interest groups are unequivocally not collective and representative of society. Patients’ perspectives therefore need to be considered in policy-relevant animal research discussions, the development of legislation and decision-making. The appropriate approach depends largely on the structure of the applicable political system.

In the past years, Switzerland has adopted its direct democratic political approach in arriving at societal decisions on the use of animals in research. In these cases, the entire population votes on the matter and it can be assumed that patients might have also expressed their position through the ballot. But legislative approaches may be different in other countries where these sorts of decisions may be made by the elected representatives of the people. But even in those systems, active patient involvement can be promoted. Generally, groups, committees and think tanks may be tasked to study the situation and make recommendations. This approach forms part of policy-relevant processes that might not involve direct involvement of all citizens but rather through the means of these advisory committees and working groups. What should be considered is actively involving patients in these bodies to represent their interests, just as other interest groups that may be represented. Patient experts should be invited to assume roles on the groups, tasks forces and working groups that work to address the societal concerns of animal research. In this regard, patients may not only be consulted by these groups when needed, but rather, they are actively involved to make direct contributions and impact in these groups. Their knowledge and contributions can again be considered in the conclusions and recommendations of these groups. Apart from their involvement in these expert groups, policymakers can also encourage and directly involve patient advocacy groups in other important deliberations that may have a significant impact on animal research policy. This will open doors for further engagement of patients in such discussions. Patients and their advocates can take leading roles in expressing their diverse opinions, knowledge of their lived experiences, and what matters most to them in biomedical research, so that society as a whole can clarify currently blurred lines for a more progressive and societally acceptable animal research policy and regulation. If needed, patients can be involved in setting priority areas for research when discussing the use of animals in specific areas of biomedical research as part of countries’ research policies and strategies.

### Involving people affected by health conditions at project levels

While the various opportunities above will be more suitable for the participation of patient representatives, patients themselves (that is, people affected by health conditions and their caregivers and family members) can also participate. They can help bridge the gap that exists between the laboratory world (which focuses on scientific validity) and the lifeworld (concerning patients' individual experiences and insights). It is understood that the promises and expectations concerning the benefits of research for patients tend to be exaggerated (Ioannidis 2004; Bennet 2013; Gomez-Marin 2021). Furthermore, waste of investment and effort has been a major challenge in the research enterprise. Biomedical research mobilizes billions of dollars and engages the skills of millions of people; however, much of the research conducted is wasted, as only a handful of discoveries make it into clinical practice (Chalmers and Glasziou 2009; Macleod et al. 2014). This inefficiency results from failures within the research production line—the choice of research questions, research designs, the conduct of research, analyses of findings, research management, and dissemination of results (Macleod et al. 2014). Addressing these issues can increase the productivity of research for society, and patient involvement can help bring about more gains from research. The participation of people affected by health conditions may make promises and expectations more precise and realistic. This means that besides patient participation on the macro and meso-levels (i.e., social and policy levels), patients may be involved at the project level, where their knowledge and experiences may help researchers shape their research approaches to strengthen the relevance and external validity of their research. Involving patients and health research consumers is possible through the mandate of research funders, whereby scientists can be required to engage patients and the public in conceiving, planning and executing their research projects (Wilson et al. 2015; Cowan 2018; Gorman and Davies 2019; den Oudendammer et al. 2019; Smits et al. 2020). Patients can therefore participate in the co-creation of research through their involvement in research planning, execution and the dissemination of findings to increase impact. To put patient involvement into practice, tools such as the *Involvement Matrix* have been developed to facilitate researchers' engagement with patients and their families to collaborate and talk about their ideas, needs and expectations at various stages of research projects (Smits et al. 2020). Moreover, patients can also participate in funding mandates by evaluating and selecting research projects that are more relevant and timely in developing therapies for conditions that affect them (Dobbs 2016; Cowan 2018; den Oudendammer et al. 2019; Costa Alencar et al. 2023). At this level, patients are able to shape the value of research by setting research priorities, defining research questions and in some scenarios, influencing the choice of animal models in animal research (Davies et al. 2024a; Davies et al. 2024b; Davies et al. 2025).

## Challenges, other concerns and way forward

In the midst of the discussion thus far, the preparedness and readiness of patients to be involved in animal research matters hold an important place in the outcomes of patient involvement initiatives. The success of this proposal depends largely on the awareness of patients about biomedical research processes and realities. How do we render patient involvement in animal research policy discussions productive and meaningful? To start with, the opportunities for patients to increase their knowledge of and get answers to their questions about biomedical research needs to be improved in order for them to contribute meaningfully to political discussions and decision-making. This is because patients may have to contribute more than their experiential knowledge about health conditions. Salience about animal research is an important aspect of societal views (Lund et al. 2012) and it should be strengthened to enhance the effectiveness of patient involvement. Researchers and research institutions can use existing engagement initiatives to raise awareness among patients about animal research. They can focus on strengthening patients’ understanding of animal research in the stages of biomedical research and therapeutic development. The knowledge patients will gain, combined with knowledge of their lived experiences in addition to their preferences, could help in making progressive political decisions about animal research in certain domains of biomedical research.

There is another significant challenge in involving patients in animal research activism and public discussions. Past initiatives of patient involvement in animal research generated controversial reactions. On the one hand, the voices of interest groups largely used patient stories in past public debates. The general approach for patient involvement at the time was based on the assumption that patients will always support animal research. Since patient testimonies were perceived to have an emotional appeal similar to those of animal activists, voices for animal research groups made use of them as part of their public relations programs (Davies et al. 2024a). Meanwhile, on the other hand, patient involvement was also constructed around anti-animal-research narratives. Through counter reactions from other parts of the patient community, other patient groups were formed in direct opposition to pro-research patient groups in the USA and UK for example (Davies et al. 2024a). Even though those initiatives are no longer active today, they are worth taking note of as we chart new paths forward in patient inclusion in public discussions and policy-relevant processes in an effort towards reaching a societal consensus. The history also tends to inform us of this possibility and how we might have to prepare to deal with it. Encountering patient groups with different positions on animal research highlights, from a glance, how divergent patients’ views on animal research can be, defeating the assumption that patients would always support animal research. Meanwhile, as stated earlier, all patients have one common interest, even if they have different views. Deliberations among themselves as well as with other stakeholders can help find a consensus regarding how biomedical research can be done to meet their needs.

From this past experience it can be feared that some interest groups may take advantage of the initiative to advance their interests which may not be necessarily that of patients. It needs to be ensured that patient involvement is constructed appropriately and there is also a need to ensure that patient involvement in these discussion does not result in unwanted exploitations by other parties, making sure that patient involvement is done legitimately. The complexities in the past took place since PPIE in health research and policy-making was not well organized at that time, and other interest groups used patient involvement in those ways. But now, what we discuss is patient involvement in the public and societal consensus processes, and this can be done more legitimately now to have legitimate impacts. In essence, it is better to make patient involvement a part of health research policy so that it can be monitored and controlled with established guidelines in order for them not to be exploited by other interest groups. A way forward can also involve engaging patients on specific topics and diseases areas regarding animal use. Above all, it is essential to ensure that the positions of patients are not assumed, and that voices using patients’ stories do not advance what (many) patients do not want.

## Conclusion

Using animals in biomedicine presents an ethical dilemma that represents a trade-off for society. It brings forth societal concerns, and society as a whole needs to collectively determine how animals are used in research from the present into the future. In this essay, we discussed the need for a collective societal approach to animal research policy while considering active patient involvement and engagement in policy-relevant processes since patients constitute a significant interest group of stakeholders and the importance of biomedical research is argued in relation to health conditions and people they affect.

Patients can be referenced when talking about health research, but it is also worth noting that all humans, at some point, are patients in need of medical interventions. Feeney disclosed that “paraplegics occasionally refer to healthy people as temporarily able bodied (TABs), as if to indicate that anyone might join the fraternity of the infirm” (Feeney 1987). This reminds us that everyone is vulnerable. A lesson that should be taken from this, in current debates and decisions about animal research as a society, reminds all involved interest groups to remember their own days of infirmity and make representative decisions accordingly. Active patient involvement offers the opportunity for this reminder when society debates and makes decisions on animal research.

Patient involvement can open a wider discussion about the clarity of the purpose of animal research for policy-relevant decision-making. Involving the perspectives of these end-users, the “so-called” beneficiaries of animal research, will add to the conversation, more refined expectations and needs that are more relevant to them and help reach a societally coherent and acceptable decision for future policies in animal research that reflects the positions of all stakeholders and interest groups.
